# Knowledge of undergraduate dental students toward orthodontic skeletal temporary anchorage devices at Kuwait University

**DOI:** 10.1186/s12909-020-02254-7

**Published:** 2020-10-06

**Authors:** Manal M. Abu Al-Melh, Abrar N. Al-Anzi

**Affiliations:** 1 Department of Developmental and Preventive Sciences (Orthodontics), Faculty of Dentistry, Health Sciences Center, Kuwait University, Kuwait City, Kuwait; 2 Department of Developmental and Preventive Sciences (Pediatrics), Faculty of Dentistry, Health Sciences Center, Kuwait University, Kuwait City, Kuwait

**Keywords:** Orthodontics, Skeletal temporary anchorage devices, Undergraduate dental students, Curriculum development

## Abstract

**Background:**

The introduction of skeletal Temporary Anchorage Devices (TADs) into orthodontics has provided orthodontists with reliable techniques of correcting anchorage problems. The objective of this study was to evaluate the knowledge of undergraduate dental students during their clinical years (fifth, sixth and seventh**-**year) at Kuwait University Faculty of Dentistry regarding the use of TADs.

**Methods:**

A printed structured questionnaire consisting of 21 questions was given to the undergraduate dental students of Kuwait University in the fifth (*n* = 22), sixth (*n* = 28) and seventh (n = 22)-years. It evaluated the level and source of information regarding orthodontic TADs.

**Results:**

The seventh-year dental students displayed the best degree of knowledge regarding the use of TADs and the clinical case management, compared with the sixth and fifth-year dental students. The significance of introducing the orthodontic TADs topic earlier in the orthodontic curriculum has been agreed upon by 42% of respondents. The total knowledge score showed a significant difference (χ2 = 40.3, *p* = 0.000), where the seventh year dental students had the best level of knowledge regarding the topic of TADs. Two-thirds (63.6%) preferred to introduce that topic during the sixth year.

**Conclusions:**

The senior (7th year) dental students had the best knowledge about the topic of TADs. Introducing the topic of TADs earlier in the undergraduate dental program will enhance the students’ case-based learning setup. Hence, the early exposure to use of TADs will improve the students’ clinical problem-solving and decision making skills during their undergraduate clinical years.

## Background

Orthodontic anchorage is commonly known as resistance to unwanted tooth movement [1]. Orthodontic skeletal Temporary Anchorage Devices (TADs), also known as miniscrews or mini-implants, are small titanium screws that are placed in the vestibular or palatal mucosa through the bone to create an independent rigid anchor unit. Moreover, TADs can be connected to nearby teeth to reinforce anchorage [2, 3]. TADs are commonly used in orthodontic treatment for a variety of reasons to enhance anchorage [4, 5]. TADs allow dental movement to be achieved at the transverse, vertical, and anterior-posterior planes without adverse effects and are sometimes necessary for optimal treatment [6, 7]. It has been shown that TADs are well accepted by the orthodontists and patients, and they are safe and effective treatment options for comprehensive orthodontic treatments [8, 9].

TADs are used for several purposes which include the retraction of anterior teeth, molar protraction or distalization, intrusion of the dentition, extrusion of impacted teeth, expansion of the arch, and orthopaedic correction of cases with maxillary deficiency. Other uses of TADs involve molar uprighting, enhancing anchorage in periodontally compromised dentition and correction of occlusal cants [10–20]. A recent study in Switzerland found that distalization against palatal mini-implants and then distalization against miniscrews were the most popular treatment plans selected by the participant orthodontists [21].

Various US programs have combined the use of TADs into their teaching curriculum and residency programs since 2005 [22]. In Kuwait, there is only one governmental dental school which is under Kuwait University authority. The dental school has a seven**-**year undergraduate program that involves 4 years of medical sciences and 3 years of dental medicine. The undergraduate orthodontic curriculum is introduced to the clinical-year students (the fifth, sixth and seventh**-**year) at Kuwait University Faculty of Dentistry (KU-FoD). Moreover, the curriculum is divided into four Orthodontics (Ortho) modules: Ortho I, Ortho II, Ortho III, and Ortho IV. The Ortho I module focuses on concepts of growth and development and orthodontic diagnosis and treatment planning, and this course is taught to the fifth year dental students. The Ortho II module and Ortho III module include topics about the diagnosis of orthodontic problems, and orthodontic mechanotherapy, and both courses are taught to the sixth year dental students. The Ortho IV module involves advanced topics in orthodontics, and it is taught to the seventh year dental students. The advanced topics taught in Ortho IV include “Failure of Eruption”, “Esthetic Orthodontics”, “Multidisciplinary Orthodontics”, “Temporary Anchorage Devices”, “Orthodontic Management of Cleft Lip/Palate and Craniofacial Deformities”, “Orthodontics and Orthognathic Surgery”, “Role of Third Molars in Orthodontics”, “Temporomandibular Disorders and Orthodontics” and “Obstructive Sleep Apnea”.

The dental curriculum at KU-FoD is one of the few programs that allow its dental students to treat limited orthodontic cases requiring simple tooth movement, and to perform a complete work-up for comprehensive orthodontic cases. This undergraduate orthodontic curriculum integrates knowledge and teaching objectives about temporary skeletal anchorage devices in the Ortho IV course during the final seventh year. To our knowledge, there are no surveys in the literature that had looked at the knowledge of undergraduate students regarding the use of orthodontic TADs as an effective treatment modality. Therefore, the current study assesses the knowledge of undergraduate dental students in their clinical years (fifth, sixth and seventh**-**year) at KU-FoD regarding the use of orthodontic TADs.

## Methods

The study population was undergraduate dental students of Kuwait University in the fifth, sixth and seventh-year. There were a total of 72 dental students: 22 students in the fifth year, 28 dental students in the sixth year and 22 dental students in the seventh year. Each dental student from the fifth, sixth and seventh years was approached and kindly asked to participate by filling-out a questionnaire and returning it after 1 week.

A printed structured questionnaire consisting of 21 questions was used for data collection. It evaluated the level and source of information regarding orthodontic TADs. The survey design was approved by the Ethical Committee of Kuwait University Health Sciences Center.

The questionnaire comprised of four main sections. The first section obtained the socio-demographic characteristics of the participants (e.g. age, gender, and year level). The second section assessed the level of knowledge of the dental students have attained during the dental school years regarding the orthodontic TADs. The third part focused on the aspects of continuing education. In the fourth part, two cases were presented to assess the students’ knowledge about clinical case management using TADs. The first case was described as a 33-year-old healthy female patient who was presented to the dental clinic with a chief complaint “I don’t like the gap between my upper and lower teeth”. Upon clinical examination and radiographic assessment, the patient was diagnosed as having a skeletal anterior open bite. Moreover, the patient rejected orthognathic surgery and any treatment option that could compromise esthetics and speech. The second case was described as a 25-year-old healthy male patient who was presented to the dental clinic with a chief complaint “I want to close the gap on the upper right side”. The patient lost his upper right first molar due to trauma.

Dental students’ knowledge was assessed by asking seven Multiple Choice Questions (MCQs) as an easy method for assessing patients’ factual knowledge, understanding, and interpretation [23]. A score of “1″ was given for the correct answer and “0″ for the incorrect or unknown answers. A total knowledge score was calculated, and it ranged from 0 to 7. It was then classified into three tertiles:

- Poor score: < 50% of the correct answers (< 3/7questions)
- Fair score: 50% — two-thirds of the correct answers (4–5/7 questions)
- Satisfactory score: >two-thirds of the correct answers (6–7/7questions)

To assess the validity of the questionnaire, a trial was conducted in which fifteen questionnaires were given to graduating dental students. This group of graduating dental students was not included in this study. Moreover, comments were received from the same group of dental students, and the questionnaire was altered accordingly.

Data were entered and analyzed using Statistical Package for the Social Science version 25.0 software (SPSS Inc., Chicago, Ill., USA). Descriptive statistics (frequencies, percentage, mean) were determined. Chi-square test was used for nominal or ordinal variables. Post hoc test, based on adjusted standardized residuals, was run to confirm where the significant differences occurred between the groups. The generated outcomes have been analyzed on the basis of *p*-value equal or less than 0.05.

## Results

All dental students in the fifth (*n* = 22), sixth (*n* = 28), and seventh (*n* = 22) year participated in this survey. The completed questionnaires were 72, which resulted in a response rate of 100%. There were a total of eight males (11.1%) and 64 females (88.9%). The male to female ratio was as follows: 2/20 in the fifth year, 1/27 in the sixth year and 5/17 in the seventh year. The majority of the students (93%) were less than 25 years of age, and they were Kuwaiti (90%). The presented socio-demographic characteristics were considered as descriptive data of the study population, and they are not considered further in the analysis of any association. Hence, this information did not affect the results of this study.

The knowledge of the responding students about orthodontic TADs is illustrated in Table 1. Regarding the definition of TADs, two-thirds of all students (68.1%) selected “mini-implants for skeletal anchorage,” and 16.7% of them did not know the answer. The vast majority of seventh-year dental students (95.5%) and sixth-year students (82.1%) got the correct answer (mini-implants for skeletal anchorage), while 22.7% of fifth-year dental students answered correctly.

**Table 1:** Level of knowledge regarding TADs among study participants

Question	5th year students	6th year students	7th year students		
	*N* = 22 N (%)	*N* = 28 N (%)	*N* = 22 N (%)	*X*^2^	*P* value
**What are orthodontic Temporary Anchorage Devices (TADs)?**					
Implants replacing a missing tooth	4 (18.2)	0	1 (1.4)	40.3	0.000**
Implants for skeletal anchorage *	5 (22.7) ^a^	23 (82.1) ^b^	21 (95.5) ^b^		
Implants for orthognathic surgeries	2 (9.1)	4 (14.3)	0		
I do not know	11 (50.0)	1 (3.6)	0		
**How well is your knowledge about orthodontic TADs?**					
Well	1 (4.5)^b^	5 (17.8)	4 (18.2)	48.9	0.000**
Fair	0	11 (39.3) ^b^	17 (77.3) ^a^		
Poor	9 (40.9)	11 (39.3)	1 (4.5)		
No knowledge at all	12 (54.5)	1 (3.6)	0		
**What is the main advantage of orthodontic TADs?**					
Tooth replacement	3 (13.7)	1 (3.6)	0	35.8	0.000**
Skeletal anchorage*	7 (31.8) ^a^	26 (92.8) ^b^	22 (100.0) ^b^		
No additional advantage	0	0	0		
I do not know	12 (54.5)	1 (3.6)	0		
**What is the most commonly used material(s) in orthodontic TADs?**					
Pure titanium*	3 (13.6) ^b^	15 (53.6) ^b^	17 (77.3) ^a^	34.8	0.000**
Titanium and stainless steel	1 (4.5)	7 (25.0)	2 (19.1)		
Titanium and copper	0	0	1 (4.5)		
I do not know	18 (81.8)	6 (21.4)	2 (9.1)		
**What are possible complications of TADs?**					
Loosening of the screw*	1 (4.5) ^b^	14 (50.0) ^b^	20 (90.9) ^a^	38.7	0.000**
Contacting a root*	0	12 (42.9) ^b^	18 (81.8) ^a^	37.7	0.000**
Pain*	4 (18.2)	9 (32.1)	6 (27.3)	4.55	0.103
I do not know	17 (77.3)	0	0	38.5	0.000**
**Can orthodontic TADs be used for growing patients in mixed dentition stage?**					
Yes*	6 (27.3)	8 (28.6)	10 (45.5)	2.10	0.349
No	16 (72.7)	20 (71.4)	12 (54.5)		
**Total knowledge score**				40.3	0.000**
Satisfactory	0	6 (21.4)	9 (40.9)		
Fair	1 (4.5)	9 (32.1)	13 (59.1) ^b^		
Poor	21 (95.5) ^a^	13 (46.4) ^b^	0		

* Correct answer, ** *p* < 0.05 = significant difference

^a-b^ values within rows with different superscript letters are significantly different (*P* < 0.05) using post hoc test

When asked about the main advantage of orthodontic TADs, more than two-thirds of all students (76.4%) chose “skeletal anchorage”. Of those students, the majority was seventh-year (100%) and sixth-year students (92%), while only a third of the fifth-year students (31.8%). A significant difference was detected (χ2 = 35.8, *p* < 0.05).

About the composition of the orthodontic TADs, around half of all dental students (48.6%) selected the correct answer “pure titanium” and one-third (36.1%) did not know the answer. Of those who selected the correct answer, there were 77.3% of the seventh-year students, 53.6% of the sixth-year students, and 13.6% of the fifth-year students. There was a significant difference between the groups (χ2 = 34.8, *p* < 0.001).

On the topic of possible complications of orthodontic TADs, most of the seventh-year students selected “loosening of the screw” (90%) and “contacting the root” (81.8%). Half of the sixth-year students selected those options (50 and 42.9% respectively). Almost none of the fifth-years students made those selections (0 and 4.5% respectively, *p* < 0.001, Table 1).

Concerning the use of orthodontic TADs for pediatric patients in the mixed dentition stage, 66.7% of all dental students would not select TADs option in children. It was shown that 27.3% of the fifth-year dental students, 28.6% of the sixth-year dental students and 45.5% of the seventh-year dental students selected “yes” for the possible use of TADs for growing patients. However, no significant difference was found between the groups.

Regarding the overall knowledge about TADs, 29.2, 38.9, and 13.9% of the participants obtained poor, fair, and satisfactory knowledge scores, respectively. Concerning the assessment of the three knowledge scores between each class, it was shown that the fifth-year dental students obtained the highest (95.5%) poor knowledge score compared to the sixth (46.6%) and seventh- (0.0%) year dental students. In addition, the seventh-year dental students acquired the highest satisfactory and fair knowledge scores (40.9 and 59.1% respectively) compared to the sixth (21.4 and 32.1% respectively) and fifth- (0.0 and 4.5% respectively) year dental students. The difference between the groups was statistically significant (χ2 = 40.3, *p* < 0.001, Table 1).

The questionnaire displayed that 65.3% of all dental students received information about orthodontic TADs during the undergraduate orthodontic program (Table 2). Moreover, 11.1% of all dental students received information on orthodontic TADs from other sources, such as social media and advertising campaigns. In addition, 70.8% of all dental students would like to receive more information about orthodontic TADs during the undergraduate program. The majority of seventh-year students and half of the sixth-year students reported that they could get more reliable information about TADs from continuing education and consultant orthodontists. Whereas the majority of the fifth-year students mentioned that they would get more reliable information only from consultant orthodontists (Table 2).

**Table 2:** TADs source of information, and aspects of continuing education according to study participants

Question	5th year students	6th year students	7th year students		
	*N* = 22 N (%)	*N* = 28 N (%)	*N* = 22 N (%)	*X*^2^	*P* value
**Did you receive any info about orthodontic mini implant during the undergraduate orthodontic program?**					
Yes	3 (13.6) ^a^	22 (78.6) ^b^	22 (100) ^b^	39.7	0.000
No	19 (86.4)	6 (21.4)	0		
**Are you receiving any info about orthodontic mini implant from other sources?**					
Yes	1 (4.5) ^b^	6 (21.4) ^a^	1 (4.5) ^b^	4.9	0.085
No	21 (95.5)	22 (78.6)	21 (95.5)		
**Would you like more info about orthodontic mini implant as a treatment modality in the undergraduate orthodontic curriculum?**					
Yes	17 (77.3)	19 (67.9)	15 (68.2)	0.64	0.727
No	5 (22.7)	9 (32.1)	7 (31.8)		
**From where would you like to get more reliable information about orthodontic mini implants?**					
Continuing education	5 (22.7)^a^	12 (44.4) ^b^	14 (63.6) ^b^	7.5	0.024
Professional journals and books	5 (22.7)	4 (14.3)	3 (13.6)	0.84	0.657
Consultants orthodontists	16 (72.7)	15 (53.6)	16 (72.7)	2.7	0.25
Internet	2 (9.1)	7 (25)	4 (18.2)	2.1	0.349
Others	1 (4.5)	0	0	2.3	0.316
**Would like to have the mini implant course introduced earlier in the orthodontic curriculum? What year?**					
Yes	15 (68.2)	13 (46.4)	14 (63.6)	2.7	0.251
In 5th year	0	0	1 (7.2)		
In 6th year	15 (100)	13 (100)	13 (92.8)		
No	7 (31.8)	15 (53.6)	8 (36.4)		
**Are you able to diagnose cases seen in your dental practice that may benefit from use of orthodontic mini implant?**					
Yes	2 (9.1)	9 (32.1)	12 (54.5)	10.5	0.005
No	20 (90.9)	19 (67.9)	10 (45.5)		

* *p* < 0.05 = significant difference

^a-b^ values within rows with different superscript letters are significantly different (*P* < 0.05) using post hoc test

The necessity of introducing the orthodontic TADs topic earlier in the orthodontic curriculum has been agreed upon by 42% of respondents. Of those, two-thirds (63.6%) of the seventh-year dental students preferred to introduce that topic during the sixth year, 46.4% of the sixth-year dental students had the same preference, and 68.2% of the fifth-year dental students selected the same choice (Table 2).

When asked about their capability to diagnose cases that may benefit from the use of orthodontic TADs, 68.1% of all students reported their inability to diagnose those cases. Of the seventh-year dental students, 54.5% of them reported the ability to diagnose cases compared to 32.1% of the sixth-year dental students and 9.1% of the fifth year dental students. The difference was statistically significant (χ2 = 10.5, *p* = 0.005, Table 2).

**Fig. 1:**
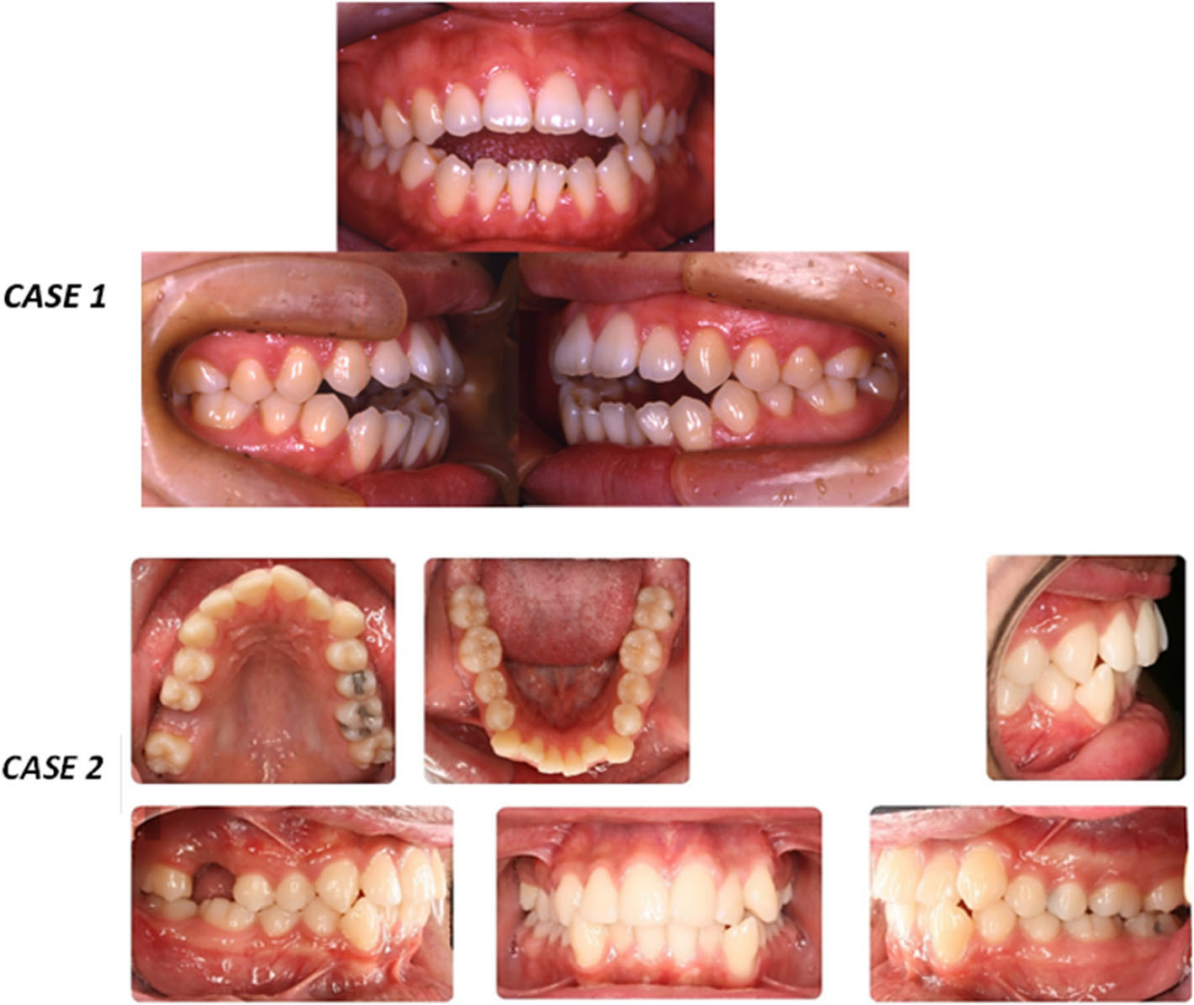
Clinical Photographs of the cases presented in the survey

Figure 1 presents two clinical cases that were used to test the participants’ knowledge regarding orthodontic management. As for case 1, the dental students were asked to select the most suitable treatment option to manage the skeletal open bite. As presented in Table 3, for the management of skeletal anterior open bite, 58.3% of all dental students selected “intrusion of posterior teeth with orthodontic TADs”, 11.1% of them chose “intrusion of posterior teeth with high pull headgear”, 8.3% of them elected “intrusion of posterior teeth with posterior bite blocks” and 22.2% of them did not how to manage the mentioned case. All seventh year students and two-thirds of sixth year students selected the treatment option “intrusion of posterior teeth with orthodontic TADs” compared to 13.6% of the fifth year students (χ2 = 51.5, *p* = 0.000). As for the ideal site for placement of the orthodontic TAD to close the anterior open bite, 30.6% of all dental students selected “around the midpalatal suture”, 8.3% of them chose “in the mandible lingually” and 61.1% of them did not have knowledge about the ideal site for placement (Table 2). More than half (59.1%) of the seventh year dental students and about one-third (32.1%) of the sixth year students selected the correct answer (around the midpalatal suture), while none of the fifth year students selected the same answer (χ2 = 18.3, *p* = 0.001, Table 2).

**Table 3:** Clinical case scenarios regarding management of an orthodontic problem and anatomical placement of TADs

Question	5th year students	6th year students	7th year students		
	*N* = 22 N (%)	*N* = 28 N (%)	*N* = 22 N (%)	*X*^*2*^	*P* value
**CASE 1: A 33-year-old patient with a skeletal open bite**					
Treatment options				51.5	.000**
Intrusion with TADs	3 (13.6)^b^	17 (60.7) ^a^	22 (100) ^a^		
Intrusion with high pull headgear	6 (27.3)	2 (7.1)	0		
Intrusion with posterior bite blocks	0	6 (21.4)	0		
Not sure what to do	13 (59.1)	3 (10.7)	0		
Ideal site for placement of TADs					
Around midpalatal suture*	0	9 (32.1) ^b^	13 (59.1) ^a^	18.3	.001**
Lingually in the mandible	3 (13.6)	2 (7.1)	1 (4.5)		
At the incisive foramen	0	0	0		
Not sure what to do	19 (86.4)	17 (60.7)	8 (36.4)		
**CASE 2: A 25 year old patient with a missing upper right molar**					
Space management				10.2	0.117
Fixed partial denture	1 (4.5)	1 (3.6)	1 (4.5)		
Prosthetic implant	8 (36.4)	14 (50.0)	12 (54.5)		
Molar protraction	5 (22.7)	8 (28.6)	9 (40.9)		
Not sure what to do	8 (36.4)	5 (17.9)	0		
Ideal site for placement of TADs aimed at molar protraction				19.8	.001**
Buccally, between upper right premolars	3 (13.6)	13 (46.4)	17 (77.3)		
Buccally, between upper centrals	0	1 (3.6) ^b^	0		
Between the upper centrals at the frenum attachment	0	0	0		
Not sure what to do	19 (86.4)	14 (50.0)	5 (22.7)		

* Correct answer, ** *p* < 0.05 = significant difference

^a-b^ values within rows with different superscript letters are significantly different (*P* < 0.05) using post hoc test

In case 2, regarding the treatment options for the space management for the missing upper right first molar, 47.2% of all students selected “a prosthetic implant”, 30.6% of them selected “molar protraction”, 4.2% of them chose “a fixed partial denture”, but 18.2% of them did not have knowledge about space management. It was demonstrated that 4.5% of the fifth-year students, 3.6% of the of the sixth-year students and 4.5% of the seventh-year students selected “fixed partial denture”. In addition, 36.4% of the fifth-year students, half of the sixth-year students and more than half of the seventh-year students selected “prosthetic implants”. Moreover, 22.7% of the fifth-year students, 28.6% of the sixth-year students and 40.9% of the seventh-year students chose “molar protraction”. However, 36.4% of the fifth-year students and 17.9% of the sixth-year students were “not sure” (Table 2). Hence, no significant difference was observed in the answers between the students in different year levels (χ^2^ = 10.2, *p* = 1.17).

As for the ideal site for placement of the orthodontic TADs for molar protraction, 45.8% of all dental students selected “buccally between the upper right first and second premolars”, 1.4% of them chose “between the upper centrals”, 52.8% of them did not have knowledge about the ideal placement site (Table 2). It has been found that 77.3% of the seventh year dental students and 46.4% of sixth year students would select (buccally between the upper right first and second premolars, whereas 86.4% of the fifth year dental students would not select it. A significant difference was found between the seventh and fifth-year students (χ^2^ = 19.8, *p* = 0.001). On the other hand, no effect of the total knowledge score of the students was shown on the answer to this question.

## Discussion

Controlling anchorage can be very challenging in orthodontic treatment. With the use of Temporary Anchorage Devices, orthodontists are now able to enhance anchorage control and avoid the undesirable side effects related to traditional orthodontics. There are several advantages to the use of TADs, including its use in a variety of clinical applications, simple placement and removal and its anchorage control for noncompliant patients [24, 25]. There is little information in the literature regarding the undergraduate dental students’ knowledge about TADs in Orthodontics. Therefore, assessing the knowledge of the students on this topic is the foundation to plan strategies on how to educate the future dentists on this promising treatment option and to help them understand the fundamentals of appropriate referrals to the orthodontists.

This study showed that, when assessing the level of knowledge regarding the use of TADs among the fifth, sixth and seventh-year dental students, the fifth-year dental students had the least knowledge about TADs compared to the sixth and seventh-year dental students. This signifies that the information about TADs is mostly attained during the seventh year. A study surveyed two groups of orthodontic providers about the use of TADs, which were graduate orthodontic residents and private practitioners. It was shown that the bulk of the residency programs (82.9%) and practitioners (69.2%) stated placing TADs in their practices [22]. It could be advantageous to integrate the topic of TADs earlier in the undergraduate dental program to allow dental students to attain adequate knowledge and the clinical experience concerning TADs.

TADs are commonly known as skeletal anchorage devices that allow difficult orthodontic movements [26]. Regarding the definition of TADS, 95.5% of the seventh year dental students answered correctly compared to the fifth year dental students (22.7%). This showed that the seventh-year dental students had the highest level of knowledge compared to the sixth and fifth-year dental students due to the adequate exposure to the TADs topic during the seventh year of the undergraduate program.

The use of TADs has several advantages, such as absolute skeletal anchorage, immediate loading, easy placement and removal [27]. In the present study, it was shown that all the seventh year dental students and almost all the sixth year dental students (92.8%) were able to correctly identify the answer to the question about the main advantage of TADs, which was “skeletal anchorage”, compared to the fifth year dental students (31.8%). This finding suggests that could be beneficial for the fifth-year dental students to receive basic information about the TADs during the fifth year.

Regarding the most commonly used material in orthodontic TADs, it is the commercially pure titanium due to its tissue biocompatibility, high corrosion resistance and lack of allergenicity [28, 29]. In this study, 77.3% of the seventh year dental students answered correctly compared to the sixth and fifth year dental students. As for the possible complications of orthodontic TADs, they can arise during placement and after orthodontic loading. Risk of trauma to the periodontal ligament or the dental root, loosening of the miniscrew and pain are some of the possible complications associated with orthodontics miniscrews [30]. This study showed that the seventh year dental students were also able to select all that applies to the question correctly compared to the sixth and fifth year dental students. Since the fifth year dental students are exposed to patients during the fifth year, it is advantageous to introduce the topic of TADs during that year to aid them patient selection, diagnosis and treatment planning.

About the possible use of TADs on growing patients in the mixed dentition stage, it was shown in the literature that TADs can show promising results in adults and children [31]. Moreover, one study showed that bone-anchored Class III protraction can be achieved with miniscrews to achieve skeletal and dentoalveolar effects in growing patients [32]. As to the possible use of TADs for growing patients, the present study demonstrated that the fifth and sixth-year dental students had the least knowledge, and less than half of the seventh-year dental students selected “yes”. Although the topic of TADs is covered during the seventh year, there is inadequate information about the possible uses of TADs for patients in the mixed dentition stage; hence, this topic should be emphasized.

When analyzing the aspect of continuing education for the dental students, this study showed that more than half of the dental students received information about TADs during the undergraduate program. This is due to the fact that the topic of TADs in mainly introduced during the seventh year as an advanced topic in orthodontics. Moreover, the undergraduate dental program focuses mainly on general dentistry, and the exposure to the topic of TADs during the dental undergraduate program is not adequate.

The majority of the dental students preferred attaining more knowledge about TADs during their undergraduate education in the dental school since they are comfortable in receiving educational information from the orthodontic mentors. Moreover, 43.1% of the dental students would like to get information from continuing education courses, and 65% of them would like to attain knowledge from consultant orthodontists during their dental school years.

Among all dental students, 42% agreed to introduce the topic of TADs earlier in the curriculum, and most of them preferred the topic to be taught during the sixth year. Therefore, it may be an advantage for dental student to have sufficient knowledge regarding TADs earlier in the curriculum. As graduating general dentists, they may not be competent to clinically treat their patients with TADs, but it is important for them to be able to inform their patients about the valid treatment options, which may include the use TADs, before referring their patients to an orthodontist.

Two clinical cases were presented to the dental students to test their ability in selecting TADs as one of the treatment options that can be presented to the patient, and to assess their knowledge about the appropriate anatomical site for TADs placement. In the first case, an adult patient was presented with a skeletal anterior open bite, and the patient rejected orthognathic surgery and treatment options that could compromise esthetics and speech. The treatment options presented to the students were mainly focused on different intrusion techniques to close the anterior open bite. In terms of orthodontic biomechanics, all options were valid for achieving intrusion of posterior teeth to close the open bite. Therefore, the question analyzed the students’ understanding of the orthodontic problem, the knowledge of biomechanics of correcting a skeletal open bite, and the ability to choose a suitable treatment option based on the information given.

It has been shown in the literature that TADs has allowed for the successful intrusion of posterior teeth with minimal need for patient compliance. Apart from the orthognathic surgery, the skeletal anchorage system for intrusion of maxillary posterior teeth is considered to be an effective technique in correcting open bite malocclusion [33]. All the treatment options presented can achieve posterior intrusion to relieve an open bite. However, given the information pertaining to the case, the high-pull headgear and posterior bite blocks treatment options may not be applicable. Considering the age of the patient, the high pull headgear would not be an appropriate option since the case is a non-growing patient, and it is unlikely that an adult patient will accept headgear for esthetic reasons. Also, posterior bite blocks could alter speech; hence, it may not be acceptable by the patient. Therefore, the use of TADs might be a more suitable option for the patient.

In the present study, it was found out that, more than half of all dental students selected “intrusion of posterior teeth with orthodontic TADs” as a valid treatment option. Also, all of the seventh-year dental students selected that treatment option, and more than half of the sixth year dental students selected the same treatment option, but a few of the fifth-year dental students selected the same option. Also, more than half of the fifth-year dental students selected “not sure what to do” for the case as biomechanics is usually taught during the sixth year. This suggests that the fifth-year dental students need to attain general information about TADs during the fifth year to assist them during clinic when treatment planning orthodontic cases.

Regarding the TADs placement site for the first case, palatal TADs placed around the midpalatal suture have been shown to be successful for the intrusion of posterior teeth [34–37]. It has been shown that more than half of the seventh year dental students selected the correct answer, but a high percentage of the fifth year dental students did not have enough knowledge to answer the question. This reinforces the fact that early exposure of dental students to the topic of TADs in the curriculum is advantageous for successful patient information. As graduating general dentists, they should be able to have adequate knowledge about the anatomical sites for successful placement of TADs.

The second case showed a 25-year-old male patient who wanted to close the gap for the missing upper right first molar. All the treatment options presented are valid ones, and the main objective of the question was to assess the students’ knowledge regarding the word “protraction”, and to assess their ability in presenting an option that involves the patient’s natural teeth rather than only the prosthetic options.

It has been shown in the literature that TADs are successful in protracting the dentition to close spaces for missing teeth [38, 39]. In the present study, a higher percentage of the dental students selected “a prosthetic implant” compared to those who elected “molar protraction”. Moreover, more than half of the seventh year dental students selected “a prosthetic implant”, half of the sixth year dental students selected the same answer and 36.4% of the fifth year dental students preferred the identical answer. This study demonstrated that dental students are more comfortable presenting treatment options that involve prosthetic work and are not competent in presenting a valid option that uses the patients’ natural dentition.

Even though the topic of TADs is taught during the seventh year, the use of TADs in the dental clinic is very seldom. This is due to lack of proper patient selection during the fifth year that may benefit from TADs. Hence, the dental students felt more comfortable selecting options that require prosthetic work, which they were heavily trained for during the clinic years. Therefore, during the fifth year, incorporating the topic of TADs and its use for dental movement, such as protraction of the dentition, would be beneficial for the dental students. This will assist them in early selection of suitable orthodontic cases and in providing treatment that requires simple tooth movement via TADs. The placement of TADs is mainly done by the orthodontist, and the dental student is allowed to observe.

Regarding the placement site of TADs in the maxilla, studies showed that interradicular placement of TADs can be successful given good cortical bone thickness and density, and that the highest bone density was found to be in permanent canine and premolar areas [40–42]. This study showed that 45.8% of all dental students selected “buccally, between the upper right first and second premolars”, but 50% of them did not have enough knowledge to answer the question. Moreover, most of the seventh year dental students answered correctly and the majority of the fifth year dental students did not know the correct answer. This re-emphasizes the importance of adequate knowledge about TADs and their anatomical placement of TADs during the early clinical years of dental school.

During the fifth year, the orthodontic curriculum focuses fundamental topics that include “concepts of growth and development”, “classification of malocclusion”, “basic orthodontic clinical examination”, “diagnosis and formulation of a problem list”, “introduction to orthodontic treatment options” and others. As a recommendation, it might be advantageous to include the topic of TADs in the “introduction to orthodontic treatment options” lecture. Basic information about TADs can be helpful for the students during treatment planning of orthodontic patients in the clinic. Moreover, as a suggestion, the topic of TADs can be incorporated in the sixth-year curriculum as it mainly focuses on mechanotherapy of orthodontic problems at the sagittal, transverse and vertical planes. Since TADs allow dental movement to be achieved at the different planes, the use of TADs can be integrated in the information given regarding the management of various orthodontic problems.

Despite the fact that this type of study has a relatively small sample size and validity problems in which respondents can over or under report specific details, the findings of the current survey established a baseline data about the knowledge of an effective modality (TADs) in the undergraduate dental program. There is a future need for determining the knowledge of dental practioners regarding the use of TADs.

## Conclusions

This study gave us insights about the knowledge associated with the use of orthodontic TADs among the dental students in clinical years at Kuwait University Faculty of Dentistry. The senior (7th year) dental students had the best knowledge because the topic of TADs was introduced to them during the final year as an advanced topic. However, since the dental students start their clinical experience by seeing patients during the fifth year, an improvement can be made in the current orthodontic curriculum by introducing the topic of TADs earlier to the dental students at the beginning of their clinical years. This will enhance their capability in selecting cases that can benefit from TADs, and broaden the scope of the possible treatment options that can be presented to the orthodontic and non-orthodontic patients.

## Data Availability

Not applicable.

## Abbreviations

TADs: Temporary Anchorage Devices
KUFoD: Kuwait University Faculty of Dentistry
Ortho: Orthodontics
